# Microstructure and Property Evolution of Pure Copper Under Asynchronous Rolling

**DOI:** 10.3390/ma18122776

**Published:** 2025-06-12

**Authors:** Shan Jiang, Long Bai, Pin Zhang, Qiang Zhu

**Affiliations:** 1 Aviation Key Lab of Science and Technology on High Performance Electromagnetic Windows, AVIC Research Institute for Special Structures of Aeronautical Composite, Ji’nan 250023, China; 18615653897@163.com (L.B.); sdzhangpin@sohu.com (P.Z.); 2 Innovation Center for Electromagnetic Functional Structure, Ji’nan 250023, China; 3 School of Materials Science and Engineering, Harbin Institute of Technology at Weihai, Weihai 264209, China

**Keywords:** asynchronous rolling, microstructural evolution, tensile properties, strain distribution, electrical conductivity

## Abstract

This study investigates the impact of asynchronous rolling on the microstructure, tensile properties, and electrical conductivity of pure copper. Pure copper sheets were subjected to asynchronous rolling with asynchronous ratios of 1.0 and 1.2, followed by microstructural analysis using electron backscatter diffraction, tensile testing, and resistivity measurements. Notable increases in ultimate tensile strength and yield strength were observed, with the sample processed at an asynchronous ratio of 1.2 showing improvements of 25.8% in tensile strength and 3.42% in yield strength compared to the sample processed at an asynchronous ratio of 1. However, an increase in strain localization and a decrease in elongation were noted, indicating a trade-off between strength and ductility. Furthermore, conductivity measurements revealed that the increase in asynchronous ratio led to a rise in defect density, resulting in a 19.96% decrease in conductivity. These findings provide valuable insights into optimizing asynchronous rolling parameters to tailor the performance of pure copper in various industrial applications.

## 1. Introduction

In the field of metal material processing, improving material properties has long been a central research objective. Traditional metalworking techniques have increasingly shown limitations in addressing the growing demand for high-performance materials [1,2,3,4]. As an effective severe plastic deformation (SPD) technique, accumulative roll bonding (ARB) has attracted considerable attention in recent years [5]. By repeatedly rolling and stacking, ARB induces ultrafine crystal structures in metals under significant plastic deformation, leading to notable improvements in strength, hardness, and corrosion resistance [6].

Asynchronous rolling is a novel technique developed as an extension of conventional ARB [2,7,8,9,10,11]. Unlike traditional synchronous rolling, asynchronous rolling involves a discrepancy in the linear speeds of the two rolls. This difference in speeds introduces additional shear deformation during the rolling process, which leads to a more uniform strain distribution within the material and a more complex deformation mechanism [12,13]. Asynchronous rolling not only refines the grain structure but also enhances the material’s anisotropy to a certain degree, offering a promising approach for producing high-performance metal materials.

Pure copper, a key non-ferrous metal, is widely known for its excellent electrical conductivity, thermal conductivity, ductility, and corrosion resistance, making it indispensable in industries such as electronics, electricity, and construction [6,14,15]. However, its relatively low strength limits its use in applications requiring higher strength [16]. The application of asynchronous rolling to pure copper holds great potential for enhancing its strength and hardness, thereby expanding its applicability while preserving its inherent outstanding properties [1,6,17,18].

Currently, research on the asynchronous rolling of pure copper remains in the early stages. While some studies have examined the effects of this process on the microstructure and tensile properties of pure copper, the deformation mechanisms, microstructural evolution, and the relationship between these properties and the microstructure still require further investigation. A deeper understanding of the characteristics of asynchronous rolling in pure copper will not only contribute to theoretical advancements in metal material processing but also provide more targeted technical guidance for industrial applications, helping to promote the broader use of pure copper in various fields.

## 2. Materials and Methods

In this study, pure copper sheets (60 mm × 15 mm × 3 mm) were procured from Baoji Boxin Metal Material Co., Ltd., Baoji, China. Prior to rolling, the copper sheets were heated to 615 °C for 70 min and subsequently furnace-cooled to ensure uniform annealing. Before initiating the asynchronous rolling process, the sheets were polished with a wire brush to remove the surface oxide layer and then ultrasonically cleaned in acetone for 3 min to eliminate any residual surface impurities. After the initial exploration, it was finally decided that we would carry out asynchronous rolling under the conditions of asynchronous rolling ratios of 1.0 and 1.2. The thickness was reduced by 50% in each pass, and the rolling was repeated three times to achieve the desired deformation.

Tensile dog-bone specimens were extracted from the processed material via wire electrical discharge machining. The tensile properties of the specimens were evaluated using a universal testing machine (Instron 5530, Instron, Norwood, MA, USA, 50 kN) at room temperature, with a strain rate of 10⁻^3^ s⁻^1^. To accurately capture the strain distribution during tensile testing, the digital image correlation (DIC) technique was employed. Prior to testing, the sample surfaces were sequentially polished using 3000-grit sandpaper to minimize surface roughness effects on tensile behavior. The polished surfaces were then coated with a matte white primer, followed by a fine toner spray, to create a speckle pattern suitable for DIC analysis. Each tensile test was performed in triplicate to ensure the reproducibility and statistical reliability of the results.

Microstructural characterization of the asynchronous rolling processed samples was conducted using electron backscatter diffraction (EBSD) in an FEI Quanta 200 FEG scanning electron microscope (SEM) with a step size of 0.2 μm. The fracture morphologies of the tensile specimens were observed using SEM (ZEISS Gemini 560, Oberkochen, Germany). Additionally, the electrical conductivity of the samples was measured using a quantum design physical property measurement system to evaluate the influence of the processing on electrical properties.

## 3. Results and Discussion

### 3.1. Microstructural Evolution

**Figure 1:**
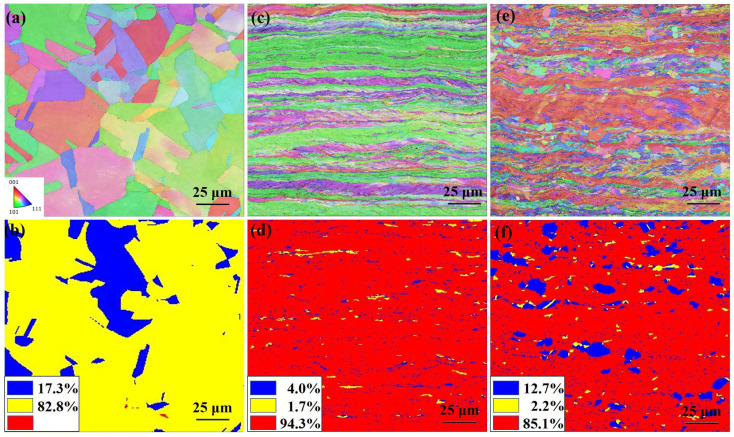
IPF and GOS of pure copper samples: (**a**,**b**) as received, (**c**,**d**) asynchronous ratio to 1, and (**e**,**f**) asynchronous ratio to 1.2.

Figure 1 presents the EBSD analysis results for the as-received sample and the samples processed via asynchronous rolling at different asynchronous ratios.

As shown in Figure 1, the microstructure of the rolled samples exhibits significant growth compared to the as-received state, indicating the influence of SPD. In contrast to the sample processed with an asynchronous ratio of 1.0, the microstructure of the sample with an asynchronous ratio of 1.2 contains not only elongated grains, which are characteristic of rolling, but also a fraction of equiaxed grains.

To further analyze the equiaxed grains in the sample with an asynchronous ratio of 1.2, grain orientation spread (GOS) was adopted to measure the local lattice distortion and recrystallization. The grains with GOS values of 0~1.5, 1.5~7, and 7~ were, respectively, identified as recrystallized grains (marked as blue), substructures (marked as yellow), and deformed grains (marked as red), as shown in Figure 1b,d,f. In the original sample, due to heat treatment, 82.8% of the grains were substructured and 17.3% of the grains were recrystallized grains. After rolling with asynchronous ratios of 1 and 1.2, deformed grains dominated, accounting for 94.3% and 85.1%, respectively, among which the substructures were not significantly different. It is worth noting that after rolling with an asynchronous ratio of 1.2, the recrystallized grains in the sample were much higher than those in the rolled sample with an asynchronous ratio of 1, increasing from 4.0% to 12.7%. This indicates that as the asynchronous ratio increases, the proportion of dynamic recovery and dynamic recrystallization increases. Finally, the bimodal grain structure shown in Figure 1e was generated, which might be due to the microstructural refinement caused by the difference in roll speed during the asynchronous rolling process [11,19,20].

**Figure 2:**
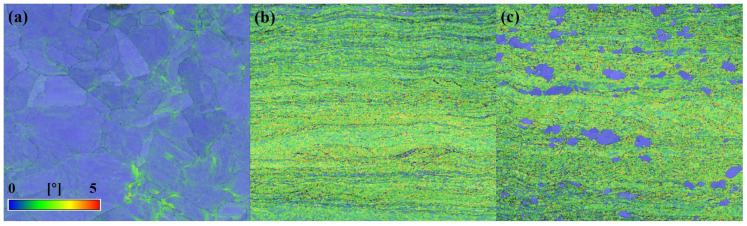
KAM of pure copper samples: (**a**) as received, (**b**) asynchronous ratio to 1, and (**c**) asynchronous ratio to 1.2.

To provide a more intuitive reflection of the accumulation of local stress and the degree of microscopic plastic deformation in the material, Figure 2 presents the distribution of kernel average misorientation (KAM) under various processing conditions [21,22]. KAM values, derived from EBSD data, serve as a critical indicator of local misorientation within individual grains.

In general, regions with higher KAM values correlate with more severe plastic deformation or higher densities of geometrically necessary dislocation (GND), which accommodate local lattice curvature induced by strain [21,23,24]. As shown in Figure 2, the as-received sample exhibits uniformly low KAM values, with most grain interiors approaching zero. Slightly elevated KAM values are found near certain grain boundaries. This pattern results from thermal processes such as recrystallization and recovery during heat treatment, which effectively reduce residual stress and dislocation density, leading to minimal internal misorientation [21,24]. In contrast, the sample processed with an asynchronous rolling ratio of 1 shows significantly increased KAM values throughout the grain interiors. This indicates substantial plastic deformation imposed during rolling, resulting in enhanced dislocation activity and lattice distortion. The relatively uniform high KAM values suggest that the plastic deformation has penetrated deep into the material, not limited to surface layers or boundaries. For the sample rolled with an asynchronous ratio of 1.2, a more heterogeneous distribution of KAM is observed. While many regions still exhibit elevated values, some areas show markedly reduced KAM. This spatial variation is likely due to the non-uniform strain caused by the speed difference between the upper and lower rolls. In localized areas, the relative motion may promote grain growth or subgrain formation, reducing the level of deformation and resulting in lower KAM values. Overall, the average KAM of the 1.2 sample is lower than that of the 1.0 sample, indicating a less uniform and reduced degree of plastic deformation.

To support these findings, the GND density is calculated through Equation (1), where $\rho_{GND}$ is the GND density, ${∆\theta }_{i}$ is the average KAM value, *μ* is the step size of EBSD measurement (0.2 μm), and *b* is the Burgers vector [21,23,25].

(1) $$\rho_{GND}=\frac{{∆\theta }_{i}}{\mu b}$$

GND density reflects the gradient of plastic strain and serves as another vital measure of internal deformation. As shown in Equation (1), the GND density in the original sample is notably low, which corresponds well with the low KAM values, confirming the stress-relieving effect of the heat treatment. Equation (1) shows a marked increase in GND density of rolling samples, that corresponds with the high KAM values and suggests substantial dislocation accumulation due to intensive plastic strain. In the sample with an asynchronous ratio of 1.2, the GND distribution is also spatially heterogeneous. Therefore, the increase in asynchronous ratio leads not only to heterogeneous KAM distribution but also to a reduction in overall GND density [21].

Both KAM and GND analyses under varying asynchronous rolling conditions provide consistent and complementary insights into the deformation behavior of the material. While the original sample remains largely undeformed, the sample with a ratio of 1 undergoes intense plastic deformation. Increasing the ratio to 1.2 introduces heterogeneous strain, resulting in localized grain growth and a general reduction in both misorientation and dislocation density [21,24].

### 3.2. Strain Distribution

**Figure 3:**
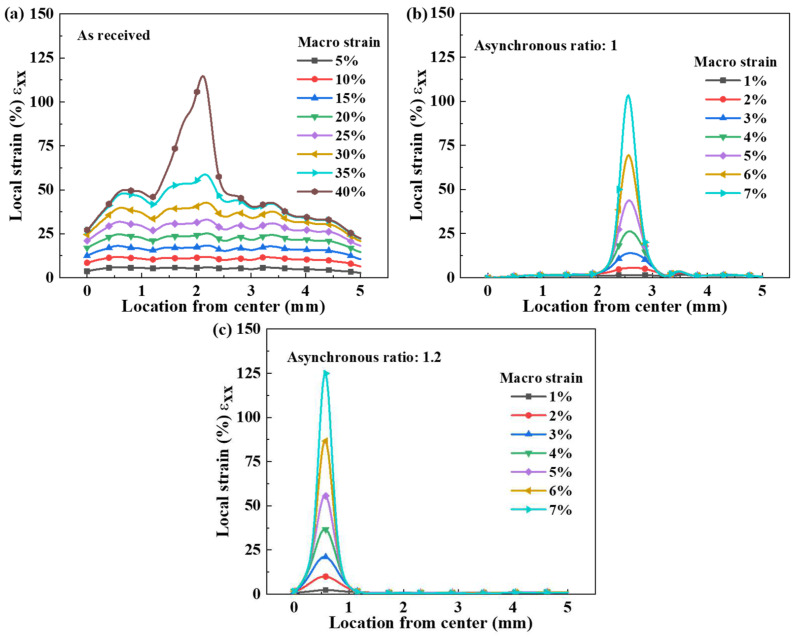
Local strain distribution along the tensile direction under different strain stages: samples (**a**) as-received, (**b**) with asynchronous ratio to 1, and (**c**) with asynchronous ratio to 1.2.

Figure 3 illustrates the linear distribution of local strain along the loading direction of the specimen during the tensile deformation.

As shown in Figure 3a, the local strain in the as-received sample gradually increases as tensile deformation progresses. When the macro strain is below 20%, the local strain distribution remains uniform, with no apparent strain concentration, indicating that the sample undergoes relatively homogeneous plastic deformation during this stage [26]. However, as the macro strain increases to 30%, the local strain in most regions of the as-received sample stabilizes around 40%, while strain concentration begins to emerge in certain areas [26].

In contrast, the local strain evolution in the rolled samples differs significantly from that of the as-received sample, as can be seen in Figure 3b,c. In these samples, strain concentration occurs immediately in specific regions upon tensile deformation, without the initial uniform plastic deformation stage observed in the as-received specimen. A comparison of Figure 3b,c reveals that the sample processed with an asynchronous ratio of 1.2 exhibits a higher local strain than the sample with an asynchronous ratio of 1.0 under the same overall strain conditions. When the macro strain reaches 7%, the maximum local strain increases from 103% to 124% as the asynchronous ratio increases, highlighting the influence of differential roller rotation on strain localization. This is mainly because asynchronous rolling causes some grains to recrystallize into equiaxed grains, enabling them to withstand higher strain concentration. However, after rolling with an asynchronous ratio of 1, most of the grains in the sample are deformed grains, and their ability to withstand strain is greatly reduced.

**Figure 4:**
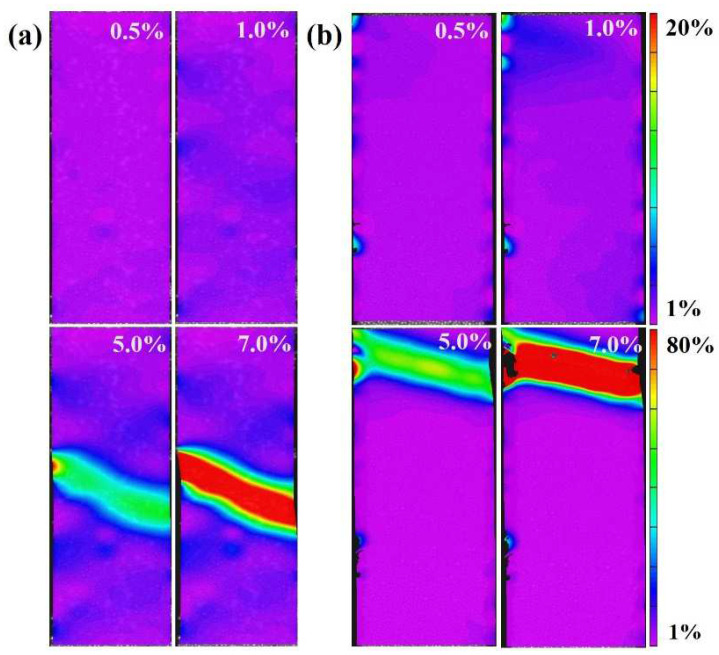
Local strain evolution during the tensile deformation: samples with (**a**) asynchronous ratio to 1, and (**b**) asynchronous ratio to 1.2.

Figure 4 illustrates the local strain distribution of pure copper samples processed via asynchronous rolling under different levels of macro tensile deformation.

At low overall strain, the local strain distribution remains relatively uniform. However, as plastic deformation progresses, localized strain concentration zones begin to emerge. When the macro strain is approximately 1.0%, the local strain remains uniformly distributed at around 1%, with no noticeable strain concentration. As the macro strain further increases, the local strain in most regions of the material remains similar to that observed at 1.0% strain. However, distinct localized strain concentration and necking regions start to develop. Moreover, under the same macro strain, the specimen processed with an asynchronous ratio of 1.2 exhibits greater local strain variation compared to the specimen with an asynchronous ratio of 1.0, as shown in Figure 3b,c. This suggests that strain concentration intensifies with increasing macro strain, indicating that a higher asynchronous ratio promotes greater strain localization during deformation.

### 3.3. Tensile Properties

**Figure 5:**
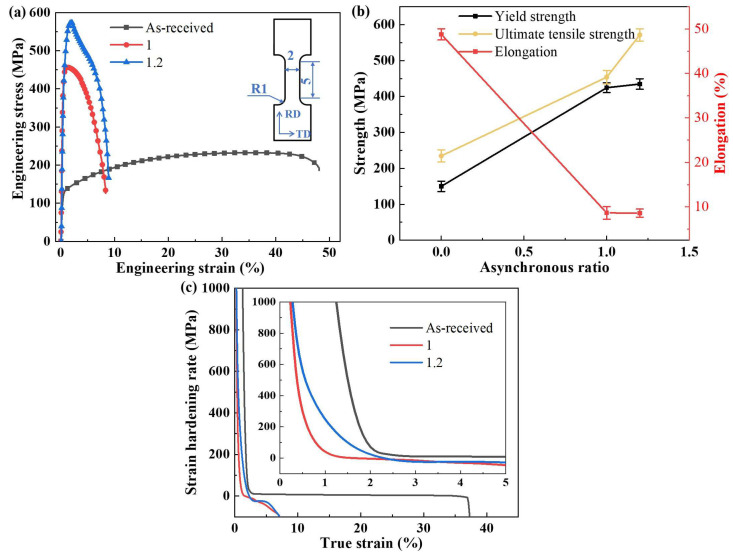
Tensile properties in different conditions: (**a**) engineering stress–strain curves under different asynchronous ratios and as-received, (**b**) variation in YS, UTS, and elongation with different asynchronous ratios, and (**c**) strain-hardening rate curves as a function of true stain.

Figure 5 presents the tensile properties of pure copper before and after asynchronous rolling at asynchronous ratios of 1.0 and 1.2. As shown in Figure 5a, the stress–strain curve of the as-received pure copper exhibits a relatively smooth profile, indicating low yield strength (YS) and ultimate tensile strength (UTS) along with substantial plastic deformation capacity over a wide strain range. Following asynchronous rolling, the curve becomes significantly steeper, reflecting a marked increase in both YS and UTS. Specifically, for the sample processed with an asynchronous ratio of 1.2, the YS increases from 420.9 MPa to 435.3 MPa, while the UTS rises from 453.9 MPa to 571.2 MPa, corresponding to increases of 3.42% and 25.8%, respectively. The strain hardening is also more pronounced, as shown in Figure 5 [12,27]. However, as the sample reaches a strain of approximately 8%, the curve declines more rapidly, indicating that its plastic deformation capacity is lower than that of the as-received sample.

Figure 5b further demonstrates that both YS and UTS increase significantly with a higher asynchronous ratio. This improvement is attributed to the generation of a large number of dislocations during the asynchronous rolling process, which entangle and impede further dislocation motion, thereby enhancing the sample’s resistance to plastic deformation and increasing its strength [12]. However, this strengthening effect is accompanied by a significant reduction in elongation, as can be observed in Figure 5a, indicating a decline in ductility. Additionally, a higher asynchronous ratio intensifies internal deformation within the sample, leading to greater stress. This elevated stress causes the sample to fracture at lower strains, further limiting its plastic deformation capacity.

The relationship between the strain-hardening rates of different specimens and the true strain is shown in Figure 5c. During the tensile deformation process, the strain-hardening rate drops sharply first, corresponding to the elastic deformation stage in Figure 5a, and the engineering stress rises sharply with the increase in strain. After the original sample undergoes the elastic deformation stage, it enters the uniform plastic deformation stage, and its strain-hardening rate remains almost unchanged. However, for the samples after rolling, due to the significant weakening of the strain-hardening ability during rolling, the stress significantly decreases after reaching the maximum stress value. Therefore, the strain-hardening rate at this stage is different from that of the original samples and shows a decrease at a lower strain.

The process of asynchronous rolling significantly enhances the strength of pure copper by introducing a high density of dislocations and promoting non-uniform deformation. However, this strength improvement comes at the expense of reduced plasticity. The observed changes in tensile properties are closely related to the microstructural evolution. In practical applications, asynchronous rolling can be utilized to improve the strength of pure copper in scenarios where high strength is required.

**Figure 6:**
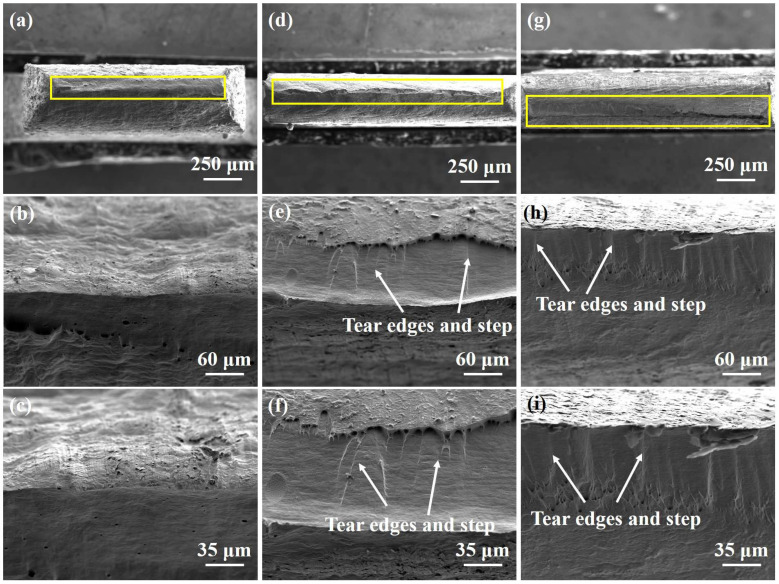
Fracture morphology in different conditions: (**a**–**c**) as-received sample, (**d**–**f**) asynchronous ratio to 1, and (**g**–**i**) asynchronous ratio to 1.2.

The fracture morphology of the samples after uniaxial tensile testing is presented in Figure 6.

As depicted in Figure 6a–c, the as-received pure copper sample exhibits excellent plasticity owing to prior heat treatment, which promotes a uniform microstructure and reduces dislocation density. As a result, the tensile fracture is characterized by a smooth and narrow necking zone, with the final fracture area appearing as a relatively sharp and raised peak [28]. The minimal presence of surface dimples indicates that the sample underwent extensive plastic deformation before failure, consistent with a typical ductile fracture mechanism. In contrast, the sample subjected to asynchronous rolling with a ratio of 1, shown in Figure 6d–f, exhibits a significantly different fracture morphology. Only a small number of dimples are visible in the fracture area, and the central fracture region is dominated by a pronounced protrusion rather than a narrowed neck. This morphological change reflects a substantial reduction in the plastic deformability due to strain hardening induced by the rolling process. The limited number of ductile features suggests that the sample has partially transitioned toward a more brittle fracture behavior [28]. Figure 6g–i further illustrate the fracture characteristics of the sample processed with an asynchronous ratio of 1.2. Although a few dimples can still be observed at the outer edge of the fracture surface, the central region is dominated by a prominent fracture protrusion with clearly discernible tear edges and step-like features. These patterns are indicative of localized shear deformation and suggest that the specimen has experienced significant embrittlement. The presence of these features implies a transition in the fracture mode: from homogeneous ductile fracture in the as-received sample to a more brittle, cleavage-like or shear-induced fracture after asynchronous rolling [28].

Overall, the evolution of fracture morphology under different rolling conditions demonstrates a clear trend: with an increasing asynchronous ratio, the fracture mechanism of pure copper gradually shifts from uniform ductile failure to localized brittle fracture [28]. This transition is closely related to the strain heterogeneity and dislocation accumulation introduced during the asynchronous rolling process, which reduces the ability of the sample to undergo uniform plastic deformation during tensile loading.

### 3.4. Electrical Properties

**Figure 7:**
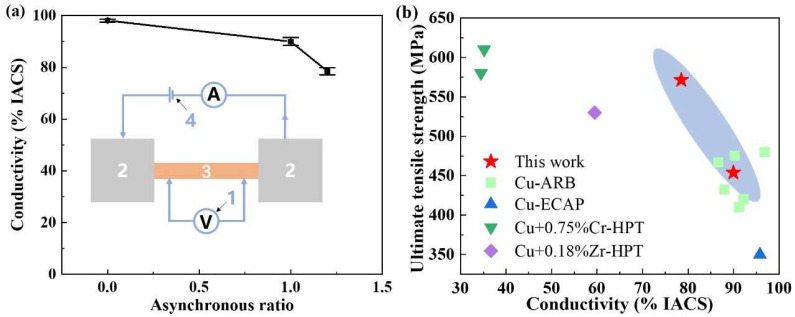
(**a**) Electrical conductivity of the samples at different asynchronous ratios; (**b**) electrical conductivity and strength [1,29,30,31].

The quantum design comprehensive physical property measurement system was utilized to assess the electrical conductivity of the samples, and the schematic diagram of the equipment is shown in Figure 7a. The initial conductivity of the pure copper sample was 98.09%. After asynchronous rolling with ratios of 1.0 and 1.2, the conductivity decreased to 89.95% and 78.51%, representing reductions of 8.31% and 19.96%, respectively. These results indicate that the asynchronous rolling process leads to a reduction in electrical conductivity, which can be attributed to the increased internal defect density caused by SPD [6,10,14].

To compare the properties of materials fabricated using different processing techniques, relevant published studies were reviewed and are summarized in Figure 7b [1,29,30,31]. The results show that after asynchronous rolling treatment, the processed samples exhibit both high strength and superior conductivity. Notably, their electrical conductivity remains significantly higher than that of samples produced by high pressure torsion (HPT) [29,30], while their strength exceeds that of materials obtained through ARB [1] and equal channel angular pressing (ECAP) [31]. These findings highlight the potential of the asynchronous rolling process for producing high-strength, high-conductivity pure copper, making it a promising approach for various industrial applications. For instance, in the field of electronic packaging, chip heat dissipation substrates, fatigue-resistant wires for aerospace use in high-frequency conductors, and precision structural components in micro-electro-mechanical systems all require high strength and high electrical conductivity in practical applications.

## 4. Conclusions

1. The asynchronous rolling process substantially improves the YS and UTS of pure copper. Specifically, at an asynchronous ratio of 1.2, the YS and UTS increased by 189.7% and 143.5%, respectively. However, the elongation decreased, indicating a trade-off between strength and ductility due to increased work hardening.
2. DIC analysis revealed that asynchronous rolling introduces strain localization early in the tensile deformation process. The degree of strain concentration increases with the asynchronous ratio, contributing to a reduction in uniform plastic deformation.
3. The conductivity of pure copper decreases with the increase in asynchronous ratio, which is primarily due to the increase in defect density resulting from asynchronous rolling. The electrical conductivity decreased by 19.96% when the asynchronous ratio was 1.2.

Although the electrical conductivity of pure copper decreases with an increasing asynchronous ratio due to the rise in internal defect density, this trade-off is accompanied by a substantial enhancement in tensile strength. The significant increase in YS and UTS demonstrates the effectiveness of the asynchronous rolling process in refining the microstructure and inducing dislocation interactions that hinder plastic deformation. This balance between strength and conductivity is crucial for practical applications, particularly in industries where mechanical durability is as important as electrical performance. By optimizing the asynchronous rolling parameters, it is possible to achieve a tailored combination of high strength and adequate conductivity, making this technique highly promising for advanced structural and functional materials in electrical and electronic applications.

## Data Availability

The original contributions presented in this study are included in the article. Further inquiries can be directed to the corresponding authors.
